# Supplementary material for: Familial Gastrointestinal Stromal Tumor Associated with Zebra-like Pigmentation
Source: Biomedicines. 2023 May 30;11(6):1590. doi: 10.3390/biomedicines11061590 (PMC10295313; doi:10.3390/biomedicines11061590)
Supplement: Supplementary file 1 [file biomedicines-11-01590-s001.zip › biomedicines-2426645-supplementary.pdf]

Supplementry Materials

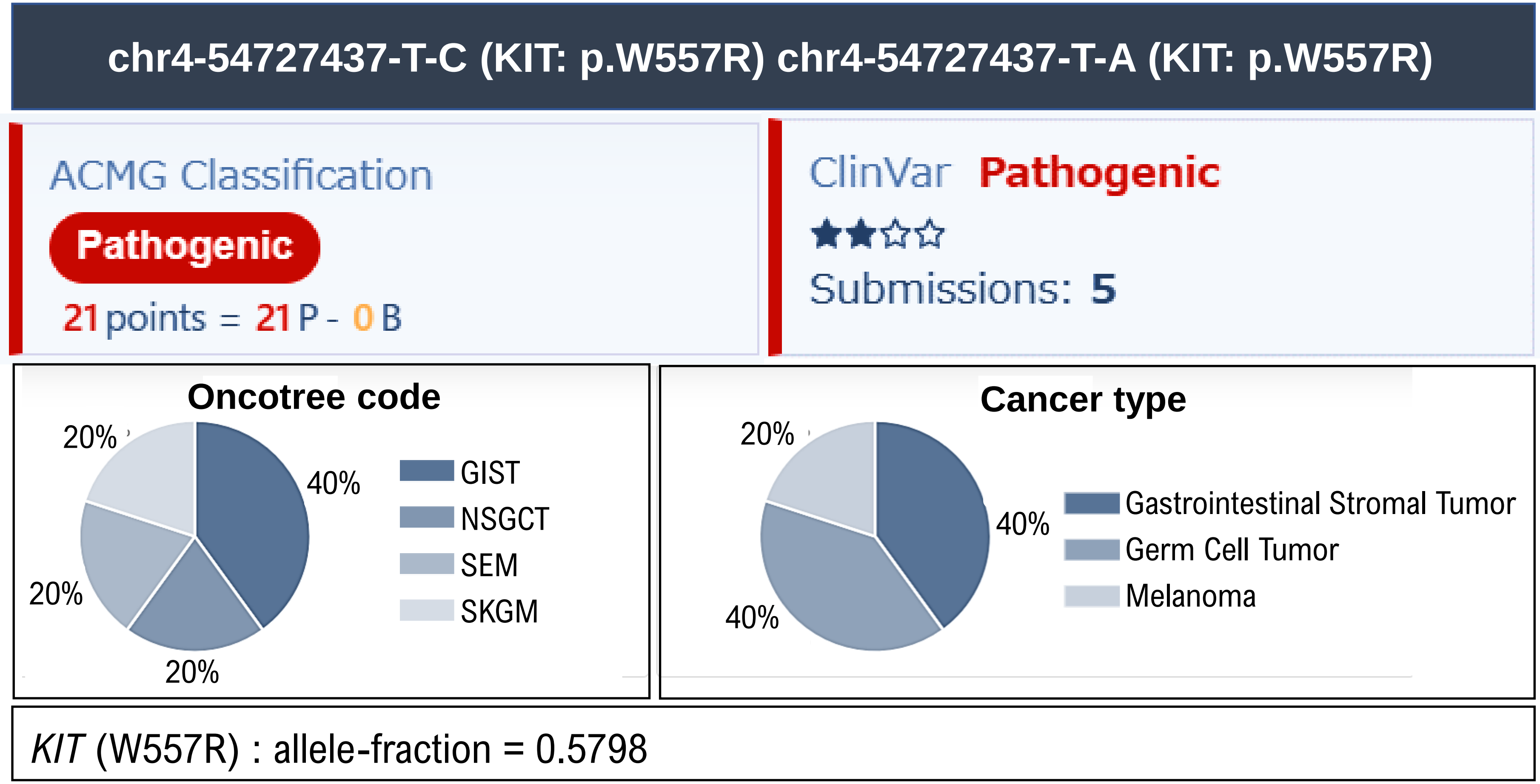

ACGM; American College of Medical Genetics and Genomics, GIST; GastrointestinalStromalTumor, NSGCT; non−seminomatous germ cell tumor, SEM; seminoma, SKGM; Shenkang granule

Figure S1. FoundationOne® CDx tissue revealed a pathogenic variant (W557R) located within exon 11 of *KIT* (allele-fraction = 0.5798), and the results were verified using the ClinVar human genome database.

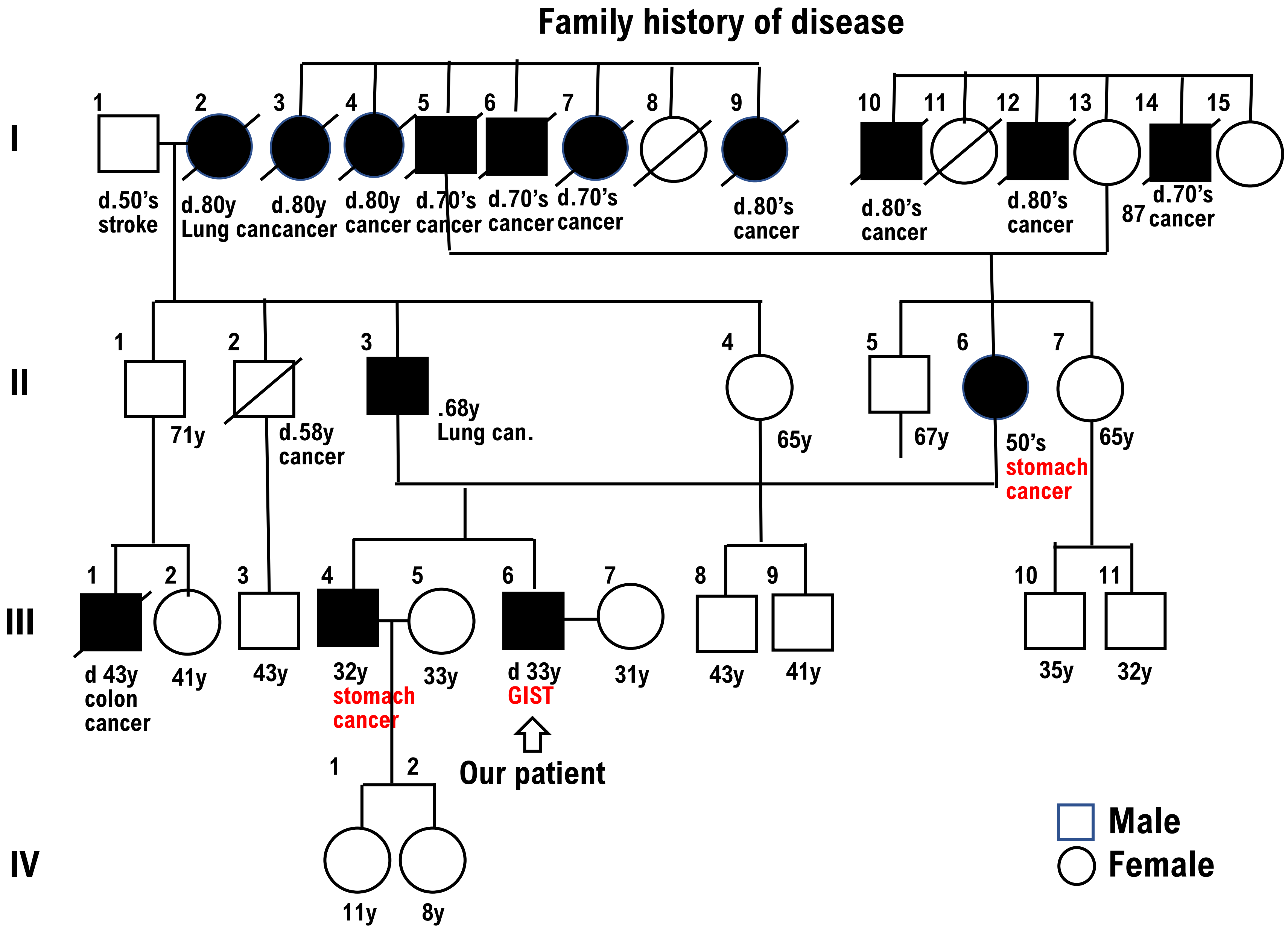

Figure S2. The patient was diagnosed with familial multinodular GIST based on his clinical findings and familial history of cancer.

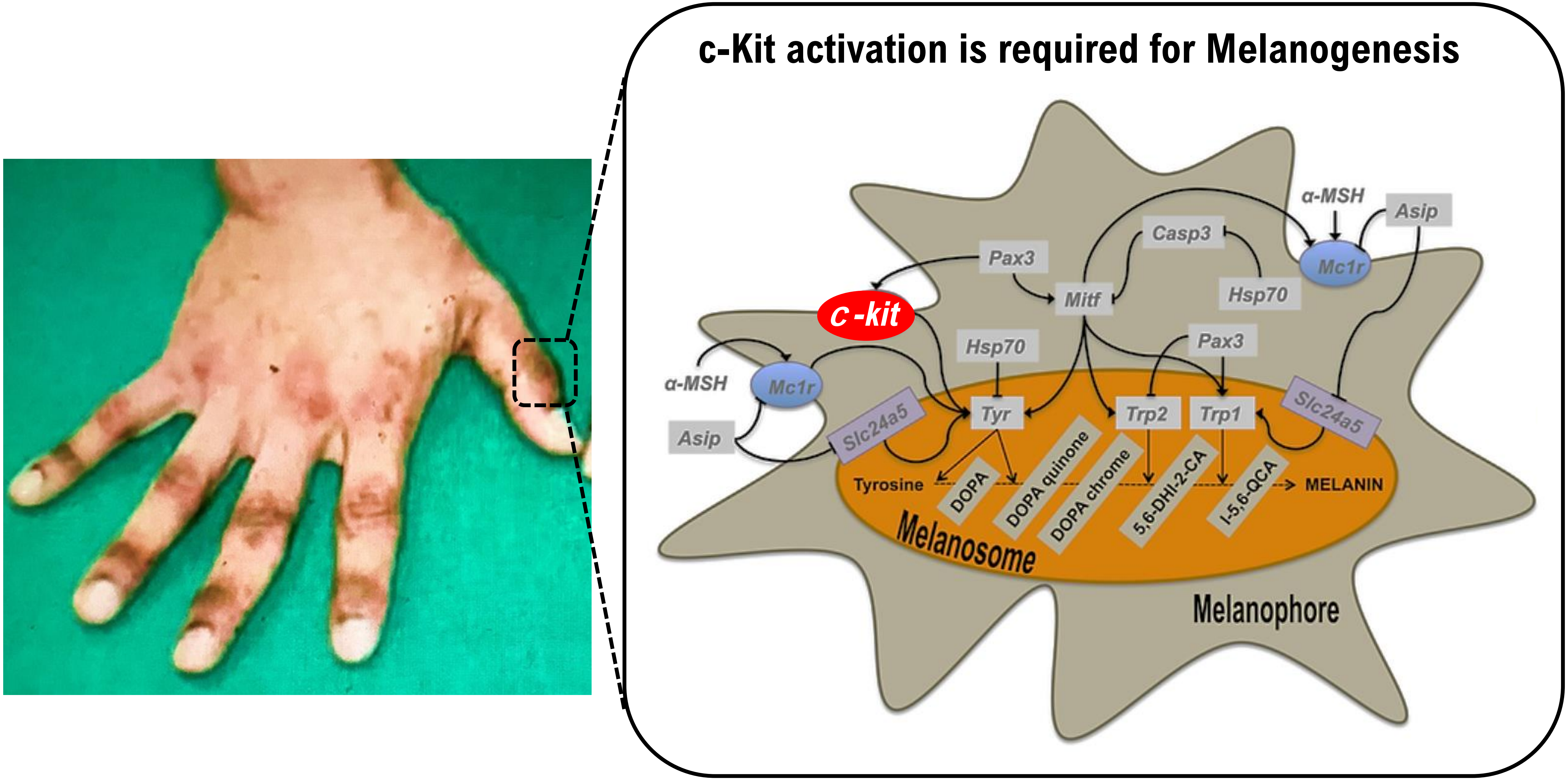

Figure S3. c-Kit activation is required for Melanogenesis.

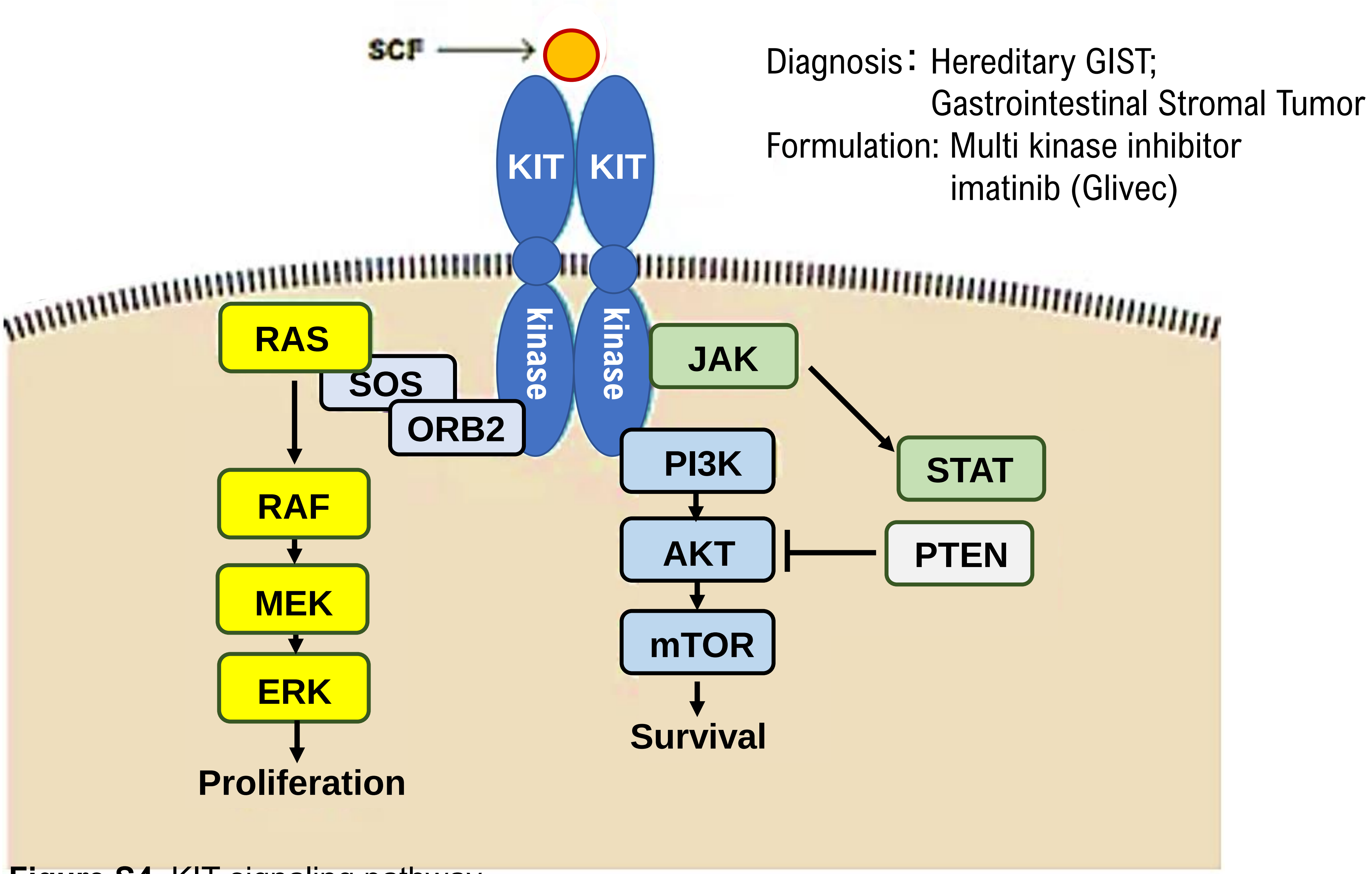

Figure S4. KIT signaling pathway.
